# Draft genome sequence of *Pantoea* sp. strain S-LA4, a potential plant probiotic endophyte isolated from the medicinal plant *Leucas aspera*

**DOI:** 10.1128/mra.00280-26

**Published:** 2026-08-14

**Authors:** Md. Shahrear Parvaj Sujon, Sharmin Islam, Soharth Hasnat, Abdullah Al Kafi, Md. Golzar Hossain, M. Nazmul Hoque, Mehede Hassan Rubel, Munima Haque, Dipali Rani Gupta, Tofazzal Islam

**Affiliations:** 1 Institute of Biotechnology and Genetic Engineering (IBGE), Gazipur Agricultural University (GAU) 198780, Gazipur, Bangladesh; 2 Department of Microbiology & Hygiene, Bangladesh Agricultural University 54492, Mymensingh, Bangladesh; 3 Department of Gynecology, Obstetrics and Reproductive Health, GAU 198780 , Gazipur, Bangladesh; 4 Agriculture Department, Noakhali Science and Technology University 378872, Noakhali, Bangladesh; 5 Department of Biotechnology, BRAC University 118864, Dhaka, Bangladesh; University of Strathclyde, Glasgow, United Kingdom

**Keywords:** Oxford Nanopore sequencing, biopesticide, probiotic, endophytic bacteria, growth-promoting

## Abstract

*Pantoea* sp. strain S-LA4 is an endophytic bacterium isolated from the leaf tissue of the medicinal plant *Leucas aspera*. The 4.93-Mbp draft genome of S-LA4 is predictive to encode several enzymes and secondary metabolites of plant growth promotion and bio-pesticidal activity, underscoring its potential for sustainable disease management in agriculture.

## ANNOUNCEMENT

*Pantoea* species are gram-negative bacteria commonly found in natural and agricultural environments, colonizing plants as epiphytes or endophytes (1). Many isolates of *Pantoea* promote plant growth through nitrogen fixation, phosphate solubilization, and siderophore production, while others act as biocontrol agents against fungal and oomycete pathogens, such as *Botrytis cinerea* and *Phytophthora infestans* (2, 3). These traits highlight the genus’s agricultural importance.

Here, we report the draft genome sequence of *Pantoea* sp. strain S-LA4, isolated from the leaves of *L. aspera* collected on May 23, 2025, in Biral Upazila, Dinajpur, Bangladesh (25.63°N, 88.55°E). Leaves of *L. aspera* were collected from multiple plants. The samples were sterilized in 70% ethanol (1 min) and 2% sodium hypochlorite (3 min), rinsed thrice in sterile distilled water, then aseptically macerated and plated on nutrient agar (Liofilchem, Italy). Distinct colonies were purified by streaking and incubated aerobically at 28°C for 48 h. A single colony was sub-cultured in nutrient broth (Liofilchem, Italy) and incubated at 28°C for 48 h, 180 rpm. Genomic DNA was extracted using the Tissue Genomic DNA Extraction HE Mini Kit (FAVORGEN Taiwan) and quantified with a Qubit 4.0 fluorometer (Thermo Fisher Scientific). Sequencing libraries prepared with ONT kits (SQK-NBD114.24, Barcoding Kit 96 V14) were sequenced on a MinION R10.4.1 flow cell, following the manufacturer’s protocol. No DNA shearing or size selection was performed. Basecalling was performed using Dorado v1.2.0 (SUP model), generated 75,425 reads in totaling 756.6-Mbp (N_50_ 11,998). Reads were quality-checked with NanoPlot v1.46.1 (4), adapters trimmed via Porechop v0.2.4 (5), and reads filtered (>1 kb) using NanoFilt v2.8.0 (6). *De novo* assembly was performed with Flye v2.9.5 (7); polished with Racon v1.5.0 (8) and Medaka v2.0.1 (9). Genome quality was assessed with QUAST v5.2.0 (10), completeness assessed with BUSCO v6.0.0 (*-l bacteria_odb10*, *-m* genome) (11), and CheckM v1.2.4 (12) using the *Pantoea* CheckM marker set. Annotation was performed with the NCBI Prokaryotic Genome Annotation Pipeline v6.10 (13) and the RAST server v2.0 (14). For all software, default parameters were used except where noted.

The draft genome comprises 4,934,719 bp in four contigs with 56.0% GC content. Annotation identified 4,475 CDSs, including 4,429 protein-coding genes and 80 tRNAs (Table 1). Taxonomic identity was assessed using the NCBI ANI workflow, showing closest similarity with “Candidatus *Pantoea astica*” (ANI 99.41%; 88.92% query coverage; 97.78% reference coverage; GCA_002434205.1). As the Taxonomy Check indicated only a genus-level match and no validated type strain was available, the organism is reported as *Pantoea* sp. strain S-LA4 (15). Predictive metabolic profiling by RAST identified genes for auxin biosynthesis (*ipdC*, *trpS*), siderophore production (*fur*, *iucD*), and ammonia assimilation (*gdhA*, *amtB*). Genes predicted to confer oxidative stress tolerance (*katG/E*, *sodA/C*) and heavy metal resistance (*arsB*, *copA/C/D*) further support its plant growth-promoting potential (16–18).

**TABLE 1:** Genomic features of *Pantoea* sp. strain S-LA4 isolated from the tissues of *L. aspera* leaves

Genome feature	S-LA4
SRA	SRR37277499
GenBank accession	JBTLRC000000000
BioSample accession	SAMN54376894
16S accession	PX285842
GenBank assembly	GCA_054482835
Number of reads	75,425
Assembled genome size (bp)	4,934,719
GC content (%)	56.0
Coverage (×)	134.0×
Genome completeness (%)	100% (BUSCO), 98.34% (CheckM)
Contamination (%)	1.68%
Number of contigs	4
N50 value (bp)	4,084,316
Genes (total)	4,587
Genes (coding)	4,429
Coding sequences (CDSs)	4,475
CDSs (with protein)	4,429
CDSs (without protein)	46
Genes (RNA)	112
rRNAs	22 (8× 5S, 7× 16S, 7× 23S)
Complete rRNAs	22 (8× 5S, 7× 16S, 7× 23S)
tRNAs	80
ncRNAs	10
Pseudogenes (total)	46
Pseudogenes (ambiguous residues)	0
Pseudogenes (frameshifted)	19
Pseudogenes (incomplete)	33
Pseudogenes (internal stop)	8
Pseudogenes (multiple problems)	12
Number of subsystems (metabolic features)	348

BAGEL4 (16) detected antibacterial bacteriocin and RiPP clusters encoding microcin and bottromycin. AntiSMASH v8.0.4 (17) predicted seven secondary metabolite clusters, with several antimicrobial activity. Together, these genomic features highlight strain S-LA4 as a likely promising candidate for biocontrol and plant growth promotion in sustainable agriculture.

## Data Availability

The whole genome sequence of *Pantoea* sp. strain S-LA4 has been deposited in NCBI GenBank (JBTLRC000000000), GenBank assembly accession GCA_054482835, and the Sequence Read Archive SRR37277499 under BioProject (Mining Bangladesh Biogold) accession PRJNA1055760. Additional annotations generated using RAST, BAGEL, and antiSMASH have been deposited in Zenodo and are accessible at https://doi.org/10.5281/zenodo.18841029.
